# The Domestic Staff—V

**Published:** 1908-09-26

**Authors:** 


### NURSING ADMINISTRATION.

### THE DOMESTIC STAFF—V.

### THE COST OF SERVICE.

Too often the cost of service is a question approached as though there were no possible method of controlling it except that of screwing down wages to their lowest figure. It has yet to be universally accepted that wages are but a small factor in determining expenditure on any department, and that the number of persons employed and the efficiency with which they discharge their duties are matters of far more importance than the rate at which they are paid. It is, however, one of the features of such a well-trained domestic staff as has been described that it offers advantages over and above money, and attracts girls who look beyond the amount of their wages. It is less costly to engage the maids young and take the trouble to train them into good servants, even though it is ultimately done for the benefit of some other institution, than to engage women who have learned in other places how not to do things properly. The junior maids can be engaged at the age of from sixteen to seventeen, and at this age it is possible in most localities to get a good choice of girls at about £12 a year. Indoor uniform will be supplied in addition. The wages will rise at the rate of £2 a year, so that at the age of twenty-one, after being four years in the service of the hospital, the maximum of £20 will be reached, when, unless there is a vacancy among the higher posts in the institution, the maid will probably leave to "better herself." In Guy's Hospital the maid is first appointed as a supernumerary or "extra," and waits her turn for a vacancy on the regular staff. During this time she is, as it were, on probation. She then becomes a junior wardmaid, and moves slowly up until she becomes a senior wardmaid. If she does well she is promoted when occasion serves to be one of the waitresses or domestic servants, and then attains to a slightly different uniform and to strings from the back of her cap. In a large institution there are various more responsible posts commanding higher wages, for which the most promising members of the domestic staff are eligible, and a good succession can be ensured of women capable in their turn of training others. A good domestic school is far-reaching in its effects, for as nurses trained in the hospital take up appointments in other institutions, they will turn to their old matron to send them housekeepers and upper servants who have been taught on good lines. Often it will happen that on leaving the institution the maid who has been through the prescribed course has so far profited that she is fit to be trained as an asylum attendant, or for some of those posts in orphanages and among the feeble-minded which need methodical and conscientious women.

A good domestic school ought undoubtedly to reduce the cost of service wherever it is established, because it enables the institution to be worked as a whole instead of in sections. To have a well-trained body of maids under the immediate control of someone who knows more about their work than they do themselves is conducive to economy in numbers, and, as has been shown many times with reference to the nursing department, it is economy in numbers which brings down the cost. Everyone is painfully aware of the slackness of ordinary institution servants. It seems sometimes as if they had been trained to do as little in the time as possible. But when under the influence of a good time-table dawdling is reduced to a minimum, and the work is despatched with a celerity which brings pleasure to the worker, the number of hands required is reduced in proportion to the time gained. There is unfortunately no standard in housework. Every matron commonly worries along with the same staff which her predecessors used, unless the general discomfort rise to such a pitch that she can persuade her board to grant her an increase in numbers. Few indeed are the administrators who have succeeded by reorganisation in reducing the numbers and at the same time increasing the efficiency of the domestic staff. We quote a few figures from Burdett's "Hospitals and Charities," showing the cost of service per bed and the number of beds per servant in some well-known hospitals:—

| — | Number of Available Beds divided among the Entire Domestic Staff | Cost of Service in Hospital and Nursing Home per Occupied Bed |
|---|---|---|
| The London Hospital ... ... | 4.4 | — |
| St. Bartholomew's Hospital ... | 6 | £5 8 |
| Guy's Hospital ... ... ... | 4 | 11 0 |
| St. Thomas's Hospital ... ... | 4.8 | 7 0 |
| St. George's Hospital ... ... | 2.6 | 17 0 |
| The Middlesex Hospital ... ... | 4.8 | 6 0 |
| St. Mary's Hospital ... ... ... | 3 | 15 0 |
| University College Hospital ... | 6 | — |
| King's College Hospital ... ... | 3.6 | 12 6 |
| The Royal Free Hospital ... ... | 3 | 14 4 |
| Charing Cross Hospital ... ... | 5 | — |
| Leeds General Infirmary ... ... | 11 | 3 6 |
| Birmingham General Hospital ... | 7 | 5 2 |
| Sheffield Royal Infirmary ... ... | 3.6 | 12 5 |
| Liverpool Royal Southern Hospital.. | 4 | — |
| Oxford, Radcliffe Infirmary... ... | 4.5 | 10 2 |
| Cambridge, Adenbrooke's Hospital.. | 4 | — |
| Edinburgh Royal Infirmary ... | 6 | 5 2 |
| Glasgow Royal Infirmary ... ... | 5 | — |
| Glasgow Western Infirmary ... | 3 | — |
| Dundee Royal Infirmary ... ... | 7.4 | 8 2 |
| The Seamen's Hospital ... ... | 8 | 6 0 |
| Great Northern Central Hospital ... | 3.4 | 15 0 |
| Norfolk and Norwich Hospital ... | 3.2 | 13 0 |
| Sunderland Infirmary ... ... | 7 | — |
| Leicester Infirmary ... ... ... | 6 | 4 9 |
| North Staffordshire Infirmary ... | 6 | — |
| Royal Devon and Exeter Infirmary.. | 6 | 10 0 |
| Cardiff Infirmary ... ... ... | 6 | — |

